# Brain Connectivity Analysis: A Short Survey

**DOI:** 10.1155/2012/412512

**Published:** 2012-10-11

**Authors:** E. W. Lang, A. M. Tomé, I. R. Keck, J. M. Górriz-Sáez, C. G. Puntonet

**Affiliations:** ^1^CIML Group, Institute of Biophysics, University of Regensburg, 93040 Regensburg, Germany; ^2^IEETA/DETI, Universidade de Aveiro, 3810-193 Aveiro, Portugal; ^3^Institute of Experimental Psychology, University of Regensburg, 93040 Regensburg, Germany; ^4^DTSTC, Facultad de Ciencias, Universidad Granada, 18071 Granada, Spain; ^5^DATC/ESTII, Universidad de Granada, 18071 Granada, Spain

## Abstract

This short survey the reviews recent literature on brain connectivity studies. It encompasses all forms of static and dynamic connectivity whether anatomical, functional, or effective. The last decade has seen an ever increasing number of studies devoted to deduce functional or effective connectivity, mostly from functional neuroimaging experiments. Resting state conditions have become a dominant experimental paradigm, and a number of resting state networks, among them the prominent default mode network, have been identified. Graphical models represent a convenient vehicle to formalize experimental findings and to closely and quantitatively characterize the various networks identified. Underlying these abstract concepts are anatomical networks, the so-called connectome, which can be investigated by functional imaging techniques as well. Future studies have to bridge the gap between anatomical neuronal connections and related functional or effective connectivities.

## 1. Brain Connectivity—What It Is All About

The functional organization of the brain is characterized by segregation and integration of information being processed. A central paradigm in modern neuroscience is that anatomical and functional connections between brain regions are organized in a way such that information processing is near optimal. Functional interactions seem to be provided by synchronized activity, both locally and between distant brain regions. Brain networks thus consist of spatially distributed but functionally connected regions that process information. Brain connectivity analysis rests upon three different but related forms of connectivity [1].

- Anatomical connectivity (AC), also called structural connectivity, which forms the *connectome* [2] through synaptic contacts between neighboring neurons or fiber tracks connecting neuron pools in spatially distant brain regions. The whole set of such fiber tracks in the brain is called white matter. On short time scales (sec, min), anatomical connections are quite persistent and stable, while for longer time spans substantial plasticity may be observed.
- Functional connectivity (FC) which is defined as the temporal dependency of neuronal activation patterns of anatomically separated brain regions. It reflects statistical dependencies between distinct and distant regions of information processing neuronal populations. Hence, it is basically a statistical concept which relies on such statistical measures as correlation, covariance, spectral coherence, or phase locking. Statistical dependencies are highly time dependent and fluctuate on multiple time scales ranging form milliseconds to seconds.
- Effective connectivity (EC) describes the influence one neuronal system exerts upon another, thus reflecting causal interactions between activated brain areas. It combines structural and effective connectivity into a wiring diagram which reflects directional effects within a neuronal network. Causality can be inferred from network perturbations or time series analysis (TSA). Techniques based on network perturbations generally need structural information as input, while TSA-based techniques, like Granger causality, may be considered model-free.

A synthesis of the latter two concepts of connectivity, mainly applied to and deduced from functional neuroimaging modalities, has been provided by Friston [3]. Functional and effective connectivity can originate, for example, from multielectrode array recordings. Both refer to abstract concepts with no immediate connection to anatomical connectivity which physically mediates such correlations. However, in recent years efforts have been undertaken to bridge the gap between these types of connectivity analysis, put forward mainly by techniques such as diffusion tensor imaging (DTI) which allow us to track fibers which form the neuronal basis for functional correlations. A recent review [4] details biophysical concepts used to model such connectivities.

In 2003, Horwitz [5] questioned the concepts of functional and effective connectivity. He argued that these notions are derived from different functional imaging modalities like functional magnetic resonance imaging (fMRI) or positron emission tomography (PET). The concept of connectivity designates the strength of interactions, whether direct or indirect, between different brain areas which locally process information. However, functional and effective connectivity are derived from quantities computed on different spatial and temporal scales, using different definitions and employing various algorithms. As long as the relation of such abstract concepts to the underlying structural connectivity between areas is not understood, comparisons across studies have to be taken with great caution. However, notice that there exists ample evidence that both concepts can be derived from the same imaging modality as well [3].

Still connectivity analysis studies created the notion of complex brain networks characterized by densely connected nodes of information processing which are distant in anatomical space and only sparsely connected via long-range connections between different functionally interacting brain regions. These network topologies reflect two basic principles underlying information processing in the brain: functional segregation and functional integration. Experimental evidence for such network topologies mainly comes from neuroimaging techniques (EEG, MEG, fMRI, PET, and SPECT) and neuroanatomical methods.

Signal transmission between distinct brain regions requires connecting fiber tracts, thus forming the structural basis of the human connectome. Diffusion-weighted magnetic resonance imaging and its variant called diffusion tensor imaging (DTI) represent the most promising approaches for fiber tracking [6]. While the former maps the diffusive motion of water molecules in the tissue returning back a single gray value per voxel only, the latter also considers the direction of diffusive motion, hence determining the second-order symmetric diffusion tensor, instead. A severe limitation of these methods, however, is their low spatial resolution. The latter can be overcome with 3D polarized light imaging (3D-PLI) [7] where the 3-dimensional course of fibers can be traced with a spatial resolution down to 100 *μ*m. Hence, 3D-PLI provides an independent evaluation of results obtained with DTI.

Brain connectivity can be quantified by encoding neighborhood relations into a connectivity matrix, whose rows and columns correspond to different brain regions. This representation lends itself to be mapped to a graphical model which provides means to quantify different topological aspects of the connectome. Graphical models represent a versatile mathematical framework for a generic study of pairwise relations between interacting brain regions. Recent years have witnessed an exponential growth of studies related to the application of graph theory to unravel characteristic features of structural, functional, and effective connectivity from neuroimaging investigations [8–10]. The most striking discovery reveals small-world properties of complex brain networks which they share with many other complex systems. A small-world topology allows a high efficiency at different spatial and temporal scales with a very low wiring and energy cost [11]. These recent discoveries indeed may indicate that the connectome is just one example of a more general universality class of complex systems observed in nature [12].

The survey is organized in the following way. First, some recent studies and reviews about experimental studies of functional connectivity are reported. This is not meant to be comprehensive, rather it should illustrate some prototypical studies in this field. Next, recent computational methods dealing with functional connectivity and some illustrative applications are collected. This is followed by a short survey of recent studies on effective connectivity. Finally, the important concept of graphical models applied to such complex brain networks as well as some applications to connectivity analysis is discussed.

## 2. Experimental Studies on Functional Connectivity

### 2.1. Experimental Studies on Static Functional Connectivity

Functional connectivity is a statistical concept which refers to statistical dependencies between voxel activity time courses. More generally, functional connectivity between two given regions is considered in terms of the temporal coherence or correlation between the oscillatory firing rate of neuronal assemblies [3]. It can be estimated through cross-correlation or covariance in the time or frequency domain, mutual information, or spectral coherence [1]. As such it reflects correlated activities within brain networks and can be deduced from neuroimaging modalities like functional magnetic resonance imaging (fMRI), electroencephalogram (EEG), magnetoencephalogram (MEG), positron emission tomography (PET), and single-photon emission computed tomography (SPECT) just to mention the most widespread techniques. The predominant technique, however, studies functional magnetic resonance imaging data and the blood-oxygen-level-dependent (BOLD) signal. In this context, functional connectivity (FC) simply refers to the temporal correlation between fluctuations in the BOLD signal of discrete anatomical regions [13]. In addition, the anatomical substrate of brain connectivity can be quantified with the help of diffusion-weighted magnetic resonance imaging tractography (dwMRIT). In practice, FC is investigated commonly by locally correlating the time course of a chosen seed voxel with the remaining voxel time courses in a voxel-by-voxel manner. This approach is biased by the actual choice of the seed voxel, however. On the contrary, spatial independent component analysis (sICA) has been shown to represent an exploratory search for global patterns of activity, thus assessing the functional connectivity of the neocortex [14].

Functional connectivity (FC), though deduced from intervoxel cross-correlations only, is nonetheless often assumed to also reflect interregional coherence of fluctuations in activity of the underlying neuronal networks in the brain. It thus is considered to refer to interregional synchrony of low-frequency fluctuations where low denotes frequencies *ν* ≤ 0.1 Hz. Note that synchrony here refers to a generalized synchrony which is defined through the mapping Ψ : *S* → *R* between a seed phase space *𝒮* and a response phase space *ℛ* such that *a*_*i*_(*t*) = Ψ(*a*_*j*_(*t*)) with some functional Ψ(⋯) and related seed *a*_*s*_(*t*) and response *a*_*r*_(*t*) activities, respectively [15]. Such interdependencies can be monitored by blood oxygenation level-dependent (BOLD) functional magnetic resonance imaging (fMRI). The latter technique utilizes spontaneous, low-frequency (*ν* ≤ 0.1 Hz) coherent fluctuations of BOLD signals to identify networks of functional cerebral connections. Since the times of Berger [16], neuroscientists believed that the brain is constantly active, even if the subjects are in a resting state condition without performing a cognitive task or receiving explicit external stimuli. These self-referential states are thought to arise from neuronal activity coherently organized in a so-called default mode network (DMN). The latter concept was first proposed by Raichle et al. [17] and summarizes an emergent body of evidence that, initiated by task-related activity, a network of brain regions, including precuneus/posterior cingulate cortex (PCC), medial prefrontal cortex (MPFC), and medial, lateral, and inferior parietal cortex, a consistent pattern of deactivation of neuronal activity is observed in these brain regions. Early studies, for example, indicated increased activity in brain areas including posterior cingulate cortex (PCC) and ventral anterior cingulate cortex (vACC) during resting state conditions [18]. Although attenuated during task performance, the DMN is active in the resting state with a high degree of functional connectivity between brain areas. This resting state activity has been termed the default mode of brain activity to denote a state in which an individual is awake and alert, but not actively involved in an attention demanding or goal-directed task [17, 19]. The coherent oscillations in the DMN exhibit characteristic frequencies below *ν* ≤ 0.1 Hz corresponding to resting state conditions, that is, without any stimulus-related cognitive tasks. This explains why originally such networks are also called resting state networks (RSNs). Meanwhile, an overwhelming evidence points to the existence of many (simultaneous) networks at rest, where the DMN is only one of them [20–23]. These early studies consistently indicated that, while performing cognitive tasks in response to external stimuli, these DMN activities are attenuated and other networks of synchronized activity emerge which adaptively reorganize themselves in a task-related and goal-oriented manner. The latter network forms the counterpart to the DMN and is often called the anticorrelated network (ACN). Both networks, the DMN and the ACN cooperate in the sense that when one of them is predominantly active, the other is less so and vice versa. Hence, brain activity in the resting state incorporates both task-negative and task-positive components. A notable exception to this general pattern of deactivation during goal-directed activity occurs in relation to tasks requiring self-referential thought or working memory where only specific DMN regions are specifically deactivated [19]. Attenuation of DMN activity has been characterized as task nonspecific meaning that the extent to which goal-directed activity influences this attenuation is dependent at least in part on cognitive load and task requirements involving functions subserved by regions within the DMN. More recently, it has been suggested that the close temporal linkage and strength of anticorrelation between the task-negative and task-positive network may allow them both to be considered components of a single default network with anticorrelated components [24]. Since the first reports about resting state activity, many different resting state networks related to vision, language, executive processing, and other sensory and cognitive domains have been identified [25]. Despite persisting skepticism as to the functional role of RSNs, Greicius et al. [25] could demonstrate that resting state functional connectivity indeed reflects the underlying structural connectivity. Note, however, that this does not implicate that there exists a simple one-to-one relationship between functional and structural connectivity. Finally, recent investigations corroborated findings that functional and structural measures of DMN connectivity have potential utility in distinguishing between mental pathologies, especially Alzheimer's dementia, and healthy controls [26, 27]. Such investigations lead to the suggestion that distorted functioning of the DMN might form the basis for many brain diseases like Autism, depressions, Parkinson's disease, Alzheimer, and related dementias. Naturally, skepticism remains against such strong assertions as it is still unclear whether functional abnormalities of the DMN are causal rather than the result of the pathology.

In a recent review, Broyd et al. [19] discuss evidence for brain dysfunction in DMN during dementias. Concerning the DMN, five key features of a DMN were discussed.

- Regional task-non-specific deactivations during goal-directed activity. Activity in the DMN becomes attenuated during task performance [28]. The more demanding the task is, the stronger the attenuation appears to be [22, 29–31]. A notable exception to this general pattern of deactivation during goal-directed activity occurs in relation to tasks requiring self-referential thought or working memory where only specific DMN regions are specifically deactivated [32, 33]. Attenuation of DMN activity thus appears to be task nonspecific. The extent to which goal-directed activity influences this attenuation is dependent, at least in part, on cognitive load and task requirements involving functions subserved by regions within the DMN [19].
- Coherence and functional connectivity within the DMN. In the context of fMRI data, functional connectivity simply refers to the temporal correlation between fluctuations in the BOLD signal of discrete anatomical regions [13]. Friston [3] first coined the term functional connectivity thereby denoting temporal coherence or correlation between the oscillatory firing rate of neuronal assemblies between two brain areas considered. Additionally, the spatial co-ordinates of the nodes within the DMN appear to substantially mirror the underlying structural connectivity between brain regions [25]. Low-frequency oscillations are likely associated with connectivity of larger-scale neuronal networks while higher frequencies are constrained in smaller networks, and may be modulated by activity in the slower oscillating larger networks [13, 34]. The functional role of Low-frequency oscillations coherent across resting state networks, and particularly the DMN, remains speculative, however.
- A Low-frequency BOLD signal. Very Low-frequency neuronal oscillations provide temporal synchrony between functionally specific and diverse regions in the DMN [24]. The coherence of such spontaneous oscillations accounts for significant variability in the trial-to-trial BOLD response observed in fMRI experiments [35]. Such coherent Low-frequency oscillations have been explored since in a variety of tasks [36] and clinical pathologies [37–39]. It was also suggested that this network of spontaneous Low-frequency activity undergoes developmental change and maturation [40–42].
- Anticorrelated task-positive and task-negative resting state networks. In the resting state, brain activity is characterized by task-positive as well as task-negative components. The latter are characteristic for the DMN as originally defined. The second network of spontaneous Low-frequency activity, the so-called task-positive network, includes the dorsolateral prefrontal cortex (DLPFC), inferior parietal cortex (IPC), and supplementary motor area (SMA). It appears to be associated with task-related patterns of increased alertness and has also been related to response preparation and selection [24, 35, 43]. The task-positive network and the DMN appear temporally anticorrelated. This reciprocal relationship between the task-positive component and DMN has been described as Low-frequency toggling between a task-independent, self-referential, and introspective state and an extrospective state that ensures the individual is alert and attentive to unexpected or novel environmental events [43, 44]. The close temporal linkage and strength of anticorrelation between the task-negative and task-positive network suggests to consider both as components of a single default network with anti-correlated components [24].
- Functions subserved by the DMN. Broyd et al. [19] further discuss some putative mechanisms for default-mode-related dysfunction in mental disorder and indicated the potential significance of altered patterns of DMN activity in subjects with mental disorder for theoretical models of psychopathology.

Greicius et al. [18] were the first to analyze the functional connectivity of a default mode network (DMN) using functional imaging. The concept of a DMN rests upon the observation of increased activity in certain brain regions, especially including the posterior cingulate cortex (PCC) and the ventral anterior cingulate cortex (vACC), during rest. The authors challenged the DMN hypothesis by studying the functional connectivity of PCC and vACC during rest, while they showed decreased activity during a working memory task. They found PCC and vACC strongly coupled with each other but also with other regions implicated by the DMN. Further, during a visual processing task, the connectivity map was found to be virtually identical to the connectivity found at rest. Finally, significant inverse correlations were found between three lateral prefrontal regions, which showed increased activity during the cognitive task, and the PCC, thus forming the corresponding ACN. This finding suggested an attenuation of DMN activity during cognitive processing and an amplification of the activity of the ACN. In summary, the default mode network (DMN) represents a consistent network of brain regions which show a high level of activity when no explicit cognitive task is performed and the participants are at rest. It is in addition defined through its reduction in activity during goal-directed behavior like passive visual fixation or resting with eyes closed. PET studies corroborated that these decreases did not arise from activations in the resting state [45]. Resting state functional connectivity networks are furthermore believed to reflect both anatomically constrained spontaneous fluctuations and state-dependent activity in fMRI studies. Gopinath et al. [46] assessed the state dependence of functional connectivity to dorsal and ventral striatum by fMRI during a resting state condition and during continuous *transcranial electrical nerve stimulation*. Results corroborate that resting state fMRI networks indeed reflect state dependent activity. Note, however, that Morcom and Fletcher [47] earlier raised serious doubts against the existence of a DMN and challenged the utility of resting state studies. They questioned the interpretability of such studies and suggested that observations made under resting state conditions have no privileged status as a fundamental metric of brain functioning.

A recent review of van den Heuvel and Hulshoff Pol [48] summarizes resting state fMRI investigations in determining functional connectivity. Possible origins of these signals are discussed as well as how functional connectivity could be related to structural connectivity, and how such connectivity patterns can be characterized and quantified through graph theoretic measures. Finally, the authors consider the role of such tools in examining alterations in functional connectivity by Alzheimer's disease and related dementias, schizophrenia, and multiple sclerosis, all of which are considered diseases with disrupted or distorted connectivities. Margulies et al. [49], furthermore, discuss the important issue of which tools are employed in analyzing fMRI recordings of the resting state. The authors review seed-based functional connectivity, independent component analysis (ICA), clustering, pattern classification, graph theory, and two local methods. They also address their underlying assumptions, methodologies, and novel applications.

Alzheimer's disease (AD) causes strong alterations of the structure and function of cerebral networks. Spontaneous brain activity is organized by synchronized activities across distinct spatial and temporal scales, thereby reflecting the complex structure of the resting state network. The latter can be studied through temporal correlations of the fMRI signals. AD-induced changes of network structure and function can thus be characterized through studying such temporal correlations at different levels of brain organization: the regional (microscopic), interregional (mesoscopic), and large-scale (macroscopic) level. Especially the PCC in the brain of patients suffering from Alzheimer's disease (AD) is vulnerable to isolation from the rest of the brain. Zhang et al. [50] examined brain regions of AD patients with connections to PCC employing resting state fMRI. Their findings demonstrated asymmetrically disrupted functional connectivity between PCC-left hippocampus, PCC-right dorsal lateral prefrontal cortex and PCC-right thalamus. In addition, regions like the bilateral visual cortex, the ventral medial prefrontal cortex, and the precuneus showed decreased functional connectivity to the PCC. However, regions in the left frontal and parietal cortex showed increased functional connectivity supporting the compensatory recruitment hypothesis. Sorg et al. [51] review studies using resting state fMRI which show that alterations in posterior areas of the default mode network (DMN), and the medial temporal lobes appear most prominent. Pronounced disturbances in neural communication appear at all spatial scales and in very early stages of the disease. *Resting state fMRI* thus seems to provide connectivity-related biomarkers which distinguish AD patients from normal controls.

White matter fibre tracts represent anatomical connectivity and provide the physical substrate for functional connectivity. In a recent review, Yo et al. [52] presented a representative selection of algorithms in use in diffusion-weighted MRI tractography (dwMRIT). The authors compared diverse methods like diffusion tensor imaging (DTI), spherical deconvolution, ball-and-stick models, and persistent angular structure along with several deterministic and probabilistic tractography algorithms on a human diffusion-weighted imaging data set. Also a novel method to quantify connectivity between brain regions has been proposed. The comparison reveals that fibre-crossing models indicate connections between a larger number of brain areas than simple diffusion tensor models. Also probabilistic tractography algorithms yield on average more connected regions with lower connectivity than deterministic models.

Combining functional and anatomical connectivity is therefore needed to reveal the relation of the former abstract concept to the physical substrate of the latter. Greicius et al. [25] test the hypothesis that resting state fMRI reflects structural connectivity rather than simply tracking BOLD signal correlations driven by nonneuronal artifacts. Diffusion tensor imaging tractography (DTIT) was combined with resting state fMRI to investigate connectivity within the DMN. The latter consisted of the medial prefrontal cortex (MPFC), the medial temporal lobes (MTL), and the posterior cingulate cortex (PCC). These regions are thought to be engaged in episodic memory processing. The fMRI connectivity maps were used to define seed regions for a DTI analysis which revealed persistent structural connections between the MTLs and the retroplenial cortex (RSC), while MPFC was connected with the PCC. Results indicate that functional connectivity deduced from fMRI measurements indeed reflects structural connections. Furthermore, the authors demonstrate that combining modalities can improve our understanding of default mode networks in the brain. Saur et al. [53] also report a combined approach but applied to the domain of language processing. Direct interactions between network nodes are identified by analyzing fMRI time series with the multivariate method of directed partial correlation (dPC). Probabilistic fibre tracking on DTI data allows to identify the most probable anatomical white matter fibre tracts mediating functional interactions. The related network topology was investigated at two levels: at the lower level of speech perception and the higher level of speech recognition. A dPC analysis revealed the functional connectivity of the related network and identified its most prominent nodes through the number of connections to other nodes. DTI tractography proved that underlying these functional connections are distinct ventral and dorsal association tracts forming the anatomical substrate which mediates these functional interactions. Hence, functional connectivity reflects structural connectivity.

### 2.2. Experimental Studies on Dynamic Functional Connectivity

Intrinsic neural networks can best be identified by measuring correlations between brain regions in resting state activity. The studies discussed above, and numerous others not mentioned here, focus on static aspects of functional connectivity. Traditionally, the analysis of resting state functional connectivity studies, employing correlation or data-driven exploratory decomposition techniques, generally assumes temporal stationarity of the recorded signals. However, recent experiments showed that functional connectivity networks may exhibit dynamic changes on short time scales. Chang and Glover [54] therefore studied the dynamics of resting state functional connectivity on the single trial level employing a time-frequency coherence analysis based on the wavelet transform. The focus was on the connectivity of the PCC, a major hub in the default mode functional connectivity network of the brain. Time and frequency-dependent variability of coherence and phase was observed between PCC and the anti-correlated network as well as for the connectivity to other nodes of the DFN. Statistical tests based on Monte Carlo simulations and a sliding window correlation technique corroborated significant scale-dependent temporal variability. It is unclear whether the observed variability in coherence and phase is due to residual noise or modulation of the cognitive state. However, it is clear that functional connectivity is not static; hence, measures of variability should be considered in addition to reporting average quantities only.

Though fMRI is a popular technique to determine functional connectivity in the brain, it is limited by its indirect nature in measuring a BOLD response rather than electrical neuronal activity directly. Brookes et al. [55] combine resting state fMRI and MEG measurements to overcome such shortcomings. With MEG, they apply envelope correlations and coherence techniques to MEG signals which were projected to source space and use beamforming to estimate functional connectivity there. Care has to be taken as cross-talk between voxels in source space may lead to spurious connectivity. Functional connectivity was estimated in sensorimotor areas using both modalities either combined or in isolation. Resulting connectivity maps showed good spatial agreement. Best results were obtained when MEG signals were filtered into the *β*-band (16–25 Hz). The method combines BOLD response-related functional connectivity with electrodynamic functional connectivity and lends credit to the hypothesis that neural activity is indeed intimately related to functional connectivity.

Resting state networks are characterized by slow fluctuations which seem to be highly structured by anatomical connections. However, the relation of these slow dynamics to fluctuating neuronal activities, particularly in the *γ*-frequency band, remains largely obscure. Slow power fluctuations of local field potentials (LFPs), as revealed by direct measurements of neuronal activities in primates, show similar large-scale correlations. Cabral et al. [56] investigated neuronal dynamics in a large-scale model of neural activity. The model consists of a structural brain network with empirically derived connections between distant brain regions and their related conductivity delays. A population of neural oscillators, performing spontaneous oscillations in the *γ*-band, were placed at each network node. The time-delayed interaction between these coupled oscillators is described by the famous Kuramoto model of phase oscillators. With realistic values for axonal conduction speed, this network exhibits slow neural activity fluctuations with patterns similar to those empirically found in functional connectivity networks. Best agreement is obtain when only a subset of nodes in the network synchronize while the global network remains desynchronized. Inside the clusters of synchronized nodes, the simulated BOLD signal is correlated between the nodes. Between clusters, positive and negative correlations are observed. The model thus explains how resting state neural activity can emerge through the interplay of local neural dynamics and large-scale network architecture.

Functionally connected regions synchronize their activities. Measuring such oscillatory dynamics requires methods with high temporal resolution like EEG, or MEG. Considering the dynamics of brain connectivity, EEG coherence is often used to measure functional connectivity in human brain [57–59]. Because of a substantial volume conduction in brain tissue and the cerebrospinal fluid (CSF), spurious coherence might overlay genuine source coherence. Similarly MEG coherence estimates are inflated at all frequencies by the field spreading between sources and sensors. Surface Laplacian EEG methods are less affected by volume conduction effects because they emphasize sources at smaller spatial scales. Hence, EEG, Surface Laplacian EEG, and MEG estimate coherences at different spatial scales and source orientations. Srinivasan et al. [60] recently confronted coherence estimates resulting from EEG, Surface Laplacian EEG and MEG recordings with simulations performed using head models derived from MRI. EEG and MEG represent noninvasive methods for identifying electromagnetic functional connectivity related to phase synchronization of pools of neural oscillators in nearby or distant brain areas. It is generally felt that simultaneous *α*-, *β*-, and *γ*-band oscillations are required for unified cognitive operations. It has been hypothesized that phase synchrony across these bands coordinates the selection and maintenance of neuronal object representations during working memory, perception, and consciousness [61]. Activity in the *α*-band is thought to reflect idling or inhibition of task-irrelevant cortical areas. But *α*-band (7–13 Hz) rhythmicity also plays an active role in mechanisms of attention and consciousness. Oscillatory activity in the *γ*-band (30–80 Hz) is thought to reflect the temporal dynamics of cortical networks and their interactions. *γ*-band activity has been found during cognitive tasks like visual perception, attention, learning, and memory as well as during processing of auditory spatial and pattern information and top-down tasks [62]. Shmuel and Leopold [63] studied the interesting question of neuronal correlates of resting state fluctuations in BOLD signals. They studied the correlation between slow fluctuations in BOLD signal and concurrent fluctuations in the underlying neuronal activity when measured locally through simultaneous fMRI and intracortical neurophysiological recordings. Correlations were most reliably detected when the neuronal signal corresponded to the local spike rate or the *γ*-band (30–80 Hz) activity of the local field potential. Patterns of correlation between voxel-by-voxel fMRI time series and neuronal activity were found to slowly traverse the cortex. The results showed that resting state fMRI-based functional connectivity between distant cortical regions can be linked to coherent slow fluctuations in the underlying neuronal signals. Spontaneous, Low-frequency (*ν* ≤ 0.1 Hz), cerebral BOLD fluctuations also show intriguing spatiotemporal patterns in functional networks which, however, are corrupted by physiological and motion confounds. Especially when studying disease-dependent changes in amplitude and spatial coherence of such Low-frequency BOLD fluctuations, such artifacts are detrimental and afford thorough preprocessing. Auer [64] reviews recent studies of the hemodynamic response to neuronal stimuli during resting state conditions and discusses their relation to physiological confounds as well as their potential for clinical diagnostic studies.

In an attempt to quantify remediation of subjects suffering from schizophrenia, Weiss et al. [65] studied accuracy and practice-related changes in graph theoretical measures indexing neural network structure and activity. MEG recordings before and after performing a tone discrimination task were used in combination with synthetic aperture magnetometry to localize brain oscillations with high accuracy. Before practice, accuracy was anti-correlated with *β*-band cost efficiency. Also in the *β*-band, sensorimotor modulations could be detected in sensorimotor cortex and the temporoparietal regions. High *γ*-band activity correlated well with sensorimotor processing following sound stimulation which elicited activity in auditory cortical areas and activity in left sensorimotor cortex before pressing a button. High *γ*-band activity in the left frontal cortex also correlated well with accuracy. After practicing for 2,5 h, sound stimulation induced increased broad-band power in the left angular gyri. In the *β*-band, improved accuracy also correlated positively with high mutual information (MI) between sensors in temporoparietal regions, whereas global cost efficiency was uncorrelated. Results suggest that practicing can induce mesoscale alterations in functional connectivity characteristic (power in certain frequency bands, MI) of task-related neural networks.

Ghuman et al. [66] report a combination of a wavelet-based method for determining phase locking in MEG data with structural MRI data providing high spatial resolution. The authors use a minimum-norm-estimate inverse solution for producing spectral functional connectivity maps starting from a predefined seed region and encompassing a broad frequency range of interest. The authors apply their method to identify interhemispheric spectral functional connectivity in a resting state auditory network in the *α*-band (7–13 Hz).

## 3. Computational Methods to Quantify Functional Connectivity

Considering prototypical studies of functional brain connectivity as discussed above, functional neuroimaging during resting state conditions seems especially interesting in that it explores spontaneous brain activity. The latter has been shown to organize itself into reproducible activity patterns. Hence, it displays structure which reflects the underlying brain architecture and carries markers of brain pathologies. An important view of modern neuroscience is that such large-scale structure of coherent activity reflects modularity properties of brain connectivity graphs. Learning such models entails two main challenges.

- Modeling full brain connectivity is a difficult estimation problem that has to face the curse of dimensionality.
- Variability between subjects, coupled with variability of functional signals between experimental trials, makes the use of multiple data sets challenging.

Concerning computational methods for functional brain connectivity studies, two broad classes may be identified, namely knowledge-based, also called supervised methods, as well as data-driven, also called exploratory or unsupervised methods. The latter can be subdivided further into decomposition methods and clustering techniques [67].

### 3.1. Knowledge-Based Computational Methods

Supervised methods afford prior knowledge about the spatial and temporal patterns of activation involved, as well as a model for the data generation process. Usually methods employ specific cognitive tasks the volunteers are supposed to perform. However, recently, they have been applied also to resting state conditions. They are widely used because of their easy implementation and straightforward interpretation. Basically, knowledge-based data analysis methods select some regions of interest (ROI) as seeds and generate a connectivity map of the human brain by determining whether other regions are functionally connected to these seeds according to predefined metrics. A convenient method to define such a metric is based on cross-correlation analysis (CCA) between the BOLD time courses of the seed region and any other brain region under consideration. Correlation is measured by the Pearson correlation coefficient *ρ*_*qs*_ given by

(1) ρqs(τ)=σqs(τ)σq·σs=〈(aq(t+τ)−〈aq(t)〉)(as(t)−〈as(t)〉)〉〈(aq(t+τ)−〈aq(t)〉)〉2〈(as(t)−〈as(t)〉)〉2,

where *τ* denotes a predefined time lag, *σ*_*i*_ denotes the variance of the neuronal activity in the query region *i* = *q* or the seed region *i* = *s*, and *σ*_*qs*_(*τ*) = 〈(*a*_*q*_(*t* + *τ*)−〈*a*_*q*_(*t*)〉)(*a*_*s*_(*t*)−〈*a*_*s*_(*t*)〉)〉 denotes the covariance of the fluctuations in neuronal activity in the query and seed regions, respectively. Functional connectivity is assumed if *ρ*_*qs*_ > *ρ*_0_ exceeds a predefined threshold *ρ*_0_. Given that the hemodynamic response function (HRF) returns to zero rather quickly (less than a minute), correlations need only to be explored for a limited number of delays which reduce the computational load of the method. In practice, CCA is often performed at zero lag which seems only justified if the signal propagation times are much less than the temporal resolution of the experimental method involved. Averaging of the pixel time courses in the seed region eliminates noise contributions to some extent. Furthermore, spatial smoothing is often employed by applying Gaussian filtering. Although commonly employed, CCA is not without problems. The HRF is known to vary between brain regions in response to vascular and metabolic factors, and even more so between subjects. Arguable assumptions about the temporal dynamics of the deoxy-hemoglobin response across the entire brain are commonly made when applying these analytical tools. Hence, zero lag CCA seems problematic, even more so as noise contributions easily create an illusion of strong correlations.

An alternative metric is based on coherence rather than correlation. The former operates in the frequency domain and is defined as

(2) $$\begin{array}{c} H_{qs}\left(\nu \right)=\frac{{\left|S_{qs}\left(\nu \right)\right|}^{2}}{S_{q,q}\left(\nu \right)S_{s,s}\left(\nu \right)}, \end{array}$$

where *S*_*ij*_(*ν*) represents either the Fourier cross-spectrum (*i* = *q*, *j* = *s*) or the Fourier power spectrum (*i* = *j* = *q*, *s*) of the related covariance functions. Of most interest is the spectral content below *ν* ≤ 0.1 Hz, as fluctuations in blood flow occur on a time scale of ten seconds, roughly. Consequently, low-pass filtering is generally employed to suppress interfering signals at higher frequencies. Coherence is invariant against frequency shifts; hence, it is insensitive to regional differences in blood flow and volume. Besides studying the magnitude of a spectrum, also its phase is of interest as phase shifts provide information about latencies between functionally connected regions. Finally, sometimes more than just one seed region needs to be considered. In such cases, it is essential to identify the specific contribution that each functional connection to only one of the regions makes. Partial correlation (PC) is a well-known technique to solve such problems efficiently by multiple regression with related control variables. By computing the correlation between the residuals of linear regressions of each of the variables of interest with the control variables, PC determines the functional connectivity between two specific regions while removing the influence of all other factors. Nowadays, the most widely used model-based method to identify functional connectivity, especially in fMRI studies, is statistical parametric mapping (SPM) [68]. It infers functional connectivity between spatially extended data by combining a general linear model (GLM) and Gaussian random field (GRF) theory. SPM uses a GLM to estimate the parameters able to explain the data and then uses GRF to resolve the multiple comparison problems in making statistically powerful inferences. Although generally employed in connection with paradigm-based designs, it has been applied also to resting-state fMRI studies lacking any designed task performance [18]. These knowledge-based approaches are all based on predefined seeds; hence, results depend on them, and different seed choices lead to different connectivity maps. Furthermore, knowledge-based methods can only study what is already known, thus missing the chance to detect unexpected connectivities not yet contained in the models employed to analyze the functional images.

### 3.2. Data-Driven Computational Methods

Exploratory data analysis techniques, predominantly decomposition and clustering techniques, represent global methods which do not rely on prior knowledge. Hence, they are able to reveal unexpected correlations in the data. These methods rely on the assumption that the brain is organized in a finite set of functional networks. Exploratory matrix factorization (EMF) techniques address such blind source separation problems by extracting, from the observations, distinct components with predefined properties from only a minimal set of constraints. Such data-driven methods deem most suitable for resting state studies exploring, beneath others, so-called default mode networks (DMN). Decomposition-based techniques such as singular value decomposition (SVD), principal component analysis (PCA), independent component analysis (ICA), and nonnegative matrix and tensor factorization (NMF/NTF) consider any observation as a linear superposition of underlying features. The latter are supposed to capture the essence of the information buried in the functional images; hence, they can also be considered feature-generating techniques. Which features are to be extracted is, however, unknown, and different methods yield different features which expose the relevant information in a more or less transparent way to the analyzer. SVD and PCA transform the functional images in a way that uncorrelated, orthogonal eigenimages result. The decomposition can be written as

(3) $$\begin{array}{c} \text{SVD}:X=U\Sigma V^{T}\\ \text{PCA}:XX^{T}=U\Sigma \Sigma^{T}U^{T}=\tilde{U}{\tilde{U}}^{T}, \end{array}$$

where **X** represents the *N* × *M*-dimensional matrix of zero mean data with all *M* functional images concatenated into *M* column vectors containing *N* ≫ *M* pixels, each. The *N* × *N*-dimensional matrix **U** represents the matrix of eigenimages of the *N* × *N*-dimensional correlation matrix **C** = **X** **X** ^*T*^, while the *M* × *M*-dimensional matrix **V** represents the matrix of eigenvectors of the corresponding kernel matrix **K** = **X** ^*T*^ **X**, and the rectangular *N* × *M*-dimensional matrix Σ contains nonnegative, real-valued singular values along its diagonal with only min⁡(*M*, *N*) singular values being different from zero. The latter correspond to the variances of the projections onto the new basis and may be used to generate a dimension-reduced representation which still preserves most of the information content. Eigenimages identify extended areas of correlated neuronal activity as long as other interfering sources of activity, like physiological noise, are not predominant. The orthogonality constraint imposed onto SVD/PCA often limits the usefulness and immediate interpretation of the *eigenimages* extracted.

In recent years, other decomposition techniques which alleviate such constraints have been considered, most notably independent component analysis (ICA) [69, 70]. The latter considers the following generic data model

(4) $$\begin{array}{c} X^{T}=MH. \end{array}$$

If the data matrix **X** ^*T*^ contains in its rows the spatial activity distribution and in its columns the different observation time points, then spatial ICA (sICA) tries to find an un-mixing matrix **M** ^†^ such that

(5) $$\begin{array}{c} H=M^{†}X^{T}, \end{array}$$

where **M** ^†^ denotes the pseudoinverse of mixing matrix **M**. Hence, **H** contains in its rows independent spatial activity distributions which are assumed to best characterize the observations, and **M** contains in its rows the corresponding weights with which each independent component contributes to the observation at any given time point. If, instead, one considers the columns of matrix **X** ^*T*^, which contain the pixel time courses of the observed functional images, then the decomposition yields independent columns of matrix **M**, corresponding to independent pixel time courses reflecting temporal variations of the observed neuronal activities, and the columns of matrix **H** then contain the corresponding weights. Hence, matrix **M** contains in its rows the temporal information, and matrix **H** contains in its rows corresponding spatial information, that is, the activity maps. Recently, also a full spatiotemporal decomposition of such two-dimensional data arrays has been discussed [71–73], but applications to functional imaging data still have to come. While PCA decorrelates second-order dependencies only, ICA tries to decorrelate all higher-order dependencies as well, thus producing statistically independent features. In practice, however, often only third- and fourth-order correlations are decorrelated as much as possible. Despite only minimal assumptions at the outset, ICA and related techniques suffer from robust and reliable techniques to estimate the unknown number of underlying independent components. Model-order selection techniques [74, 75] like minimum description length (MDL), Akaike information criterion (AIC), Bayes information criterion (BIC), and so forth are known to mostly overestimate the number of components. An additional difficulty is to measure the independence of the extracted components reliably, especially in higher dimensions where mutual information as the most reliable indicator of independence is hard to estimate. In fact, blind source separation presumes the existence of the sources sought for, as well as their number, while EMF tries to decompose any given set of observations into components as independent as possible. Hence, a certain degree of independence is always achieved when EMF techniques are applied. In that respect, a third concern is about independence in itself, as it is by no means clear why there should exist independent networks of neuronal activity distributions in the brain at all. Hence, other paradigms like dependent component analysis (DCA) which allows for dependencies in groups of components, which however are independent from other groups, might become attractive to the functional imaging community as well. As sparseness entails independence or at least uncorrelatedness, EMF techniques might be pushed towards yielding sparse components instead of independent ones [76]. Although the existence and extraction, with EMF techniques, of meaningful component networks of neuronal activity related to key information processing steps in the brain are still a matter of debate, denoising and artifact removal can be achieved quite reliably with such techniques. Hence, EMF techniques can also be employed as proper preprocessing methods even for later processing using seed-based procedures. An especially demanding and still not satisfactorily solved problem is an EMF analysis across groups of subjects. As EMF techniques miss any natural ordering of the components extracted, identifying corresponding components across a group of volunteers is still a matter of debate and methodological-development [77]. Several approaches have been put forward so far encompassing template matching [26, 78], temporal concatenation of individual data sets registered from a group of subjects [79], dual, that is, spatial followed by temporal, regression of group level data sets [80, 81], and back-reconstruction of group level data sets decomposed individually with ICA [82]. However, most of these approaches entail template matching at a certain stage of analysis which renders their outcome strongly dependent on the quality of the templates established beforehand.

Considering EMF as an unsupervised data analysis tool and the number of extracted components as an unconstrained parameter of the model, these techniques may also be categorized as clustering methods which achieve an unsupervised partitioning of the data set into subsets according to a predefined metric or nonmetric [83] similarity measure. In case of EMF, observations are projected onto the new basis system generated for a new representation of the data set, and these projections are grouped according to their size. Other clustering techniques employed in functional imaging encompass hierarchical clustering, partitional clustering, and spectral clustering often accompanied by multidimensional scaling, gaussian mixture models, bootstrapping and bagging [84–89]. Hierarchical clustering, whether agglomerative or divisive, can achieve any predefined number of clusters, where the appropriate number of clusters can be decided upon after the partitioning process. With partitional clustering, the number of clusters needs to be fixed before the clustering starts. Typically the number of clusters is optimized by minimizing intracluster variance to obtain homogeneous clusters according to some appropriate and problem-dependent measure of homogeneity. Spectral clustering first performs an eigendecomposition of the Kirchhoff matrix of the underlying graph and afterwards clusters the data on the basis of the resulting eigenvectors [90]. Similar to EMF techniques, the drawback of all clustering algorithms is the unknown number of cluster into which the data set would naturally decompose. Recently, probabilistic methods have been proposed to overcome this almost ubiquitous model-order selection problem [91, 92] proposing techniques which are known as automatic relevance detection (ARD).

Closely related to clustering are classification problems, especially when functional connectivity is to be compared between certain disease states and their normal counterparts. The latter comparison is especially interesting when images are acquired under resting state conditions. With specific stimuli presented, such multivoxel pattern analysis has been named brain reading [93, 94]. An essential prerequisite of such multivariate approaches is a robust and reliable feature extraction stage where appropriate features are generated, a subset of which is then used for classification purposes to identify specific mental states of the brain. All classifiers need to be trained with preclassified activity patterns. Subsequent testing including cross-validation [95] provides measures for the generalization ability in terms of specificity, selectivity, and accuracy of the classifier employed. Measures like receiver-operating characteristics (ROCs curves) and the related area under the curve (AUC) are generally used to measure the performance of the classifier. Classifiers employed most frequently are linear Fisher discriminant analysis (LDA), linear support vector (SVM), or nonlinear Kernel machines or tree classifiers like random forests (RF). Generally, the success of any classifier rests upon the quality and appropriateness of the features it is provided with to perform the discrimination task. Given proper features, often simple linear classifiers achieve high accuracy, while with improper features even the most sophisticated classifiers fail to achieve good results. Applying SVMs, a technique called recursive feature elimination (RFE-SVM) [96], can be applied to find the most discriminate subset of features for the classification problem at hand. A similar goal can be achieved by computing the Gini index [97] of an RF classifier which provides an importance measure for each feature relative to the discrimination task considered.

### 3.3. Some Applications of Computational Methods

#### 3.3.1. Computational Studies on Static Connectivity

Assessing functional connectivity from neuroimaging recordings essentially follows two strategies: seed based versus ICA based. The two methodologies can be combined estimating temporal correlations with a specified seed voxel or small region of interest and spatially independent components (sICs). Independent component analysis (ICA) and related exploratory decomposition techniques set out to approximate any observed activity distribution **X** as a superposition **X** ^*T*^ ≈ **M** **H** of a number of underlying activity distributions **H**, called features, which characterize nearly independent subnetworks engaged in cognitive information processing. Seed-based FC measures were shown recently to be the sum of ICA-derived within-network connectivities and between-network connectivities [98]. Both methodologies are thus intimately related and provide essentially similar information. However, other than voxel-based statistical methods, exploratory matrix decomposition techniques like ICA or principal component analysis (PCA) are not easily generalized across a group of volunteers.

Recent evidence from several neuroimaging studies suggests that the human brain has a modular hierarchical organization which resembles the hierarchy depicted by different ICA model orders (the number of columns of the mixing matrix **M**). Resting state networks (RSNs) can be reliably and reproducibly detected using independent component analysis (ICA) at both individual subject and group levels. Elseoud et al. [99] recently hypothesized that functional connectivity between group differences measured with ICA might be affected by model-order selection. They investigated differences in functional connectivity using so-called dual regression as a function of ICA model order. The results showed that the detected disease-related differences in functional connectivity alter as a function of ICA model order. Especially high model orders showed an increased risk of false positives that needs to be overcome. The findings of Elseoud et al. suggest that multilevel ICA exploration of functional connectivity enables optimization of sensitivity, that is, the number of true positives (TP) versus the sum of the number of TP and false negatives (FN), to brain disorders. Exploratory methods for discovering unknown connectivities, in general, must control their false discovery rate (FDR = TP/(TP + FP)) induced by random variations in the data. Li et al. [100] describe a method for graphical models which allows to control the FDR of the conditional dependence relationship which a graphical model encodes. A group analysis of an fMRI study on Parkinson's disease revealed an effective control of the FDR by the method proposed.

Estimating functional or effective connectivity relies on the correlational or causal structure of activity distributions in distant brain areas. Such activity patterns, however, are subject to intra- and intersubject variations. Hence, it is generally of interest to identify sources of variation for fMRI connectivity. Rogers and Gore [101] performed an empirical comparison of various sources of variation within an fMRI study of functional connectivity. More specifically, they estimated functional and effective connectivity in the motor cortex based on intersubject variation in task activation level, within subject variation in task-related response, and within-subject residual variation after removal of task effects. Though, for two different task conditions, results showed different interregional correlation coefficients, all three measures yielded qualitatively similar results concerning condition differences in connectivity. Hence, within-subject and between-subject results can be usefully compared. Furthermore, correlations in residual time series indicate that residuals do not simply correspond to resting state activities. Rather, they reflect variations which also underly steady-state performance.

Varoquaux et al. [102] report for the first time a cross-validated model of spontaneous brain activity. The study describes the brain functional connectivity structure at the subject level as a multivariate Gaussian process and introduce a new strategy to estimate it from group data by imposing a common structure on the graphical model in the population. The authors show that individual models learned from fMRI data using this population prior generalize better to unseen data than models based on alternative regularization schemes. They, furthermore, use the estimated graphical model to explore the large-scale characteristics of functional architecture and show for the first time that known cognitive networks appear as the integrated communities of a functional connectivity graph.

#### 3.3.2. Computational Studies on Dynamic Connectivity

Sofar functional connectivity has been discussed only in a static perspective. A dynamic system's perspective needs to deal with time dependencies of functional connectivities and has to consider studies of functional network features across a broad range of frequencies. Hence, instead of employing matrix factorization techniques, functional connectivity can also be modeled in the frequency domain using multivariate autoregressive models (MVAR). Traditionally, such estimates based on MVAR models neglect instantaneous effects. Erla et al. [103] discuss the impact of including zero-lag interactions and evaluate performance differences to traditional MVAR models using the directed partial coherence (dPC). Simulations with instantaneous interactions generated misleading connectivity patterns resulting from traditional MVAR analysis. The authors concluded that EEG data, where instantaneous effects cannot be neglected, need to be analyzed with extended MVAR models to properly elucidate direction and strength of the interactions among EEG rhythms. Haufe et al. [104] discuss a new method based on MVAR models to assess functional brain connectivity in EEG/MEG signals which is called sparsely connected source analysis (SCSA). SCSA represents EEG signals as a linear mixture of correlated sources within an MVAR model. It estimates the demixing simultaneously with the MVAR model parameters while avoiding overfitting by imposing the Group Lasso penalty. A data-driven model of functional connectivity then arises from extracting appropriate levels of cross-talk between the extracted sources.

Dynamic neuronal activity can be characterized locally by employing EEG or MEG recordings. However, the large-scale structure of synchronized cortical networks remains poorly characterized still. Palva et al. [105] combine simultaneous EEG and MEG recordings across all frequency bands to estimate the architecture of phase-synchronized networks of neuronal oscillators employing an inverse modeling based on a minimum norm estimate. Stimuli were derived from a visual working memory maintenance task. Time- and frequency-dependent interregional phase synchrony was estimated from single-trial phase differences. The latter were derived from cortical patches covering the entire cortical surface. Graph theoretical measures were applied to characterize networks specific for the various frequency bands, and salient differences could be detected between the *δ*/*θ*-band (3–6 Hz), the *α*-band (7–13 Hz), the *β*-band (16–25 Hz) and the *γ*-band (30–80 Hz). Especially *α*- and *β*-band networks showed a more pronounced clustering tendency and small-world characteristics but had a less pronounced global efficiency than *δ*/*θ*- and *γ*-band networks. Further, *α*- and *β*-band networks exhibited a truncated power-law degree distribution indicating a memory-load-dependent scale-free small world structure with densely connected core-like clusters. Hence, dependent on the cognitive state, synchronized dynamic functional connectivity networks appear different in different frequency bands and might support distinct functional roles.

Deco et al. [106] recently concentrated on emerging concepts of the dynamics of complex brain networks. They reviewed three large-scale neural system models of the neocortex which emphasize the prominent role of local dynamics, signal transmission delays, and noise to the emerging RSNs. The authors suggest that the emergence and disappearance of resting state patterns of activity reflect explorations of functional network configurations around a stable anatomical skeleton. In a related review, Deco and Corbetta [107] point towards the decisive role of the dynamics in the network and advocate the view that resting state activity networks are functionally organized as competing systems, both at rest and during task performance. In anticorrelated networks, noise-driven transitions between multistable cluster synchronization states drive the dynamics in these networks. Multi-stable states are considered to emerge because of transmission delays between brain regions. The latter are modeled as coupled oscillator systems. Dynamics in large-scale networks are such that different functional subnetworks are maintained in a state of enhanced competition. Computational studies suggest that the latter can be either stabilized or excited by small modulations of either sensory or internal signals.

#### 3.3.3. Computational Studies on the Development of Functional Connectivity

Another important aspect which recently came into the focus of current research is the development of functional connectivity in the developing brain. Fair et al. [108] studied the development of the functional organization of brain networks. They combined resting state fMRI, graph theoretic analysis, community detection, and spring-embedding visualization techniques to analyze four distinct networks previously identified. They show that the developing brain is characterized by a general decrease in correlation strength (segregation) between anatomically close brain regions balanced by an increase in connection strength (integration) of anatomically distant regions. Graph theoretical measures, especially small-world properties like clustering coefficients and average path lengths, turn out to be similar in local subnetworks compared to large-scale global networks. Community detection shows a modular organization with stable communities within the graphs which are distinctly different in early (children) and later (youngsters) stages of development. This implies that similar information processing problems are solved in divergent way during maturation of the human brain. Similar conclusions were drawn later by Vogel et al. [109] who reviewed resting state fMRI studies of brain development in humans. As a general principle, a segregation and integration mechanism appears whereby predominantly anatomically localized interactions in children develop towards more distributed interactions spanning longer cortical distances. Thus, brain maturation occurs via segregation of functionally connected local regions and integration of functionally connected distant regions finally forming large-scale networks of disparate, highly connected subnetworks which themselves are sparsely interconnected. The importance of specific interregional functional connections forming in the developing brain, driven by genetic as well as environmental factors, is further discussed by Shannon et al. [110]. The authors studied resting state networks in impulsive juveniles versus normal controls by fMRI. They showed that, in normal controls, motor planning regions are correlated with networks associated with spatial attention and executive control. To the contrary, in impulsive teenagers, motor planning regions are strongly correlated with the default mode network (DMN) which is associated with spontaneous, self-referential cognition. A subsequent study of the functional connectivity of the developing brain over a large age span corroborated these findings and showed a strong correlation between the characteristics of the changing functional connectivity structure and emerging impulsivity patterns. Results suggest that impulsivity of the offender population is caused by a delayed but typical maturation of the brain rather than a distinct abnormality. Smyser et al. [111] review recent studies of neonatal brain development by fMRI. Especially problems concerning the nature, location, and timing of changes during brain development need to be studied further. The authors conclude that optimal methods for functional connectivity MRI data acquisition and analysis of neonatal infant populations need to be defined still. Further, appropriate schemes of interpretation and translation of results from fMRI connectivity studies remain unknown and need to be explored.

### 3.4. Effective Connectivity

Effective connectivity is directed and dynamically changes according to a given context or a task performed. Therefore, one important aspect of effective connectivity analysis is to dig out the directionality of causal influences. If an observation of temporal fluctuations in the neuronal activity in one brain region allows to better predict future temporal fluctuations in the neuronal activity in another region, then the former region is said to influence the latter. Understanding brain connectivity generally follows two different routes: dynamic causal modeling (DCM) [112] models effective connectivity (EC) by studying how activities in distinct brain areas affect each other, while Granger causal modeling (GCM) [113] looks for correlations in the activity of several regions and thus builds upon functional connectivity (FC). Motivations behind both methodologies as well as controversies about extracting causal interactions from BOLD measurements are discussed in Friston, [114–116]. 

Zhou et al. [117] considered combining PCA with Granger causality to study directional influences between functional brain regions within an fMRI connectivity analysis employing both simulated as well as human fMRI data sets. PCA was applied as preprocessing to reduce the number of fMRI time series. The authors show that thereby more energy, and information-related features can be preserved than using only averaged activity values of the ROIs. Granger causality can then be applied to the extracted principal components to further study effective connectivity. Results of an analysis of emotion task-induced activities, localized in the anterior cingulate cortex, the inferior frontal sulcus, and the amygdala show that directional influences between these regions could be resolved and between-regions causalities could be better represented.

While these methods do not entail temporal aspects, Rajapakse et al. [118] describe a probabilistic framework, based on dynamic Bayesian networks (DBNs), for estimating effective connectivity among activated brain areas from fMRI data sets. Bayesian networks are often used to learn the structure of effective connectivities at any given time. It thus represents a snapshot of the dynamically changing effective connectivities with no temporal information. The latter can be deduced from fMRI time series data by modeling them using Markov chain methods. Simulations based on synthetic fMRI data show good correspondence of the resulting effective connectivity structures to Granger causality mapping [119]. Brain connectivity is thus described in statistical terms, and temporal characteristics, encoded in the voxel activity time series, are explicitly taken into account. Such dynamic Bayesian networks were used in the aforementioned work to represent interactions between regions, and Markov random fields (MRFs) serve to represent contextual dependencies within functional images. Brain activation and effective connectivity are estimated simultaneously without the need for any *a priori* model of connectivity.

Roebroeck et al. [120] also concentrate on a dynamical system perspective and review work on causal time series analysis. Their review focuses on dynamic causal analysis of fMRI data to infer brain connectivity from a time series analysis and dynamical systems perspective. Causal influence is expressed in the Wiener-Akaike-Granger-Schweder (WAGS) tradition, and dynamical systems are treated in a state space modeling framework. The nature of the fMRI signal is reviewed with emphasis on the involved neuronal, physiological, and physical processes and their modeling as dynamical systems. In this context, two streams of development in modeling causal brain connectivity using fMRI are discussed: time series approaches to causality in a discrete time tradition and dynamic systems and control theory approaches in a continuous time tradition. This review closes with discussion of ongoing work and future perspectives on the integration of the two approaches.

Contrary to anatomical connectivity, effective connectivity flexibly depends on contexts and tasks. Battaglia et al. [121] show how dynamic effective connectivity can emerge from transitions in the collective organization of synchronized neuronal activity. Mesoscale network motifs of interacting cortical areas are studied analytically and via simulations. Computations are based on extended random neural networks with nodes corresponding to either spiking neurons or simply rate units. A causal analysis of the time series of model neuronal activity is performed. It reveals that different dynamical states generated from an identical structural connectivity motif correspond to distinct effective connectivity motifs. Directionality in effective connectivity can emerge from symmetry breaking despite reciprocal underlying structural connections. It is shown also that the dynamics of effective connectivity control both the efficiency and directionality of information transfer through fixed structural connectivity motifs. These results nicely demonstrate that dynamic interactions between neuronal activities in distant brain areas provide “the basis for the self-organized control of this *communication-through-coherence*, making thus possible a fast *on-demand* reconfiguration of global information routing modalities.”

Despite the potential usefulness of the concept of effective connectivity, it remains a source of constant concern and ongoing discussion, mainly because of the temporal blurring induced by the hemodynamical response.

## 4. Graphical Models of Brain Networks

Graph-theoretical concepts experience increasing attention in recent years in characterizing static and dynamic structures of complex brain networks [122, 123]. Graphical models provide means to characterize complex brain connectivity networks, so-called brain graphs [9, 10]. Graphs may be constructed for anatomical networks as well as for functional networks. Thus, they offer a theoretical framework to describe the structural and functional topology of system-wide brain networks. In recent years, a wealth of studies have considered graph theory [9, 124] as an appropriate tool to characterize and analyze patterns of neuronal activity during task performance or under resting state conditions. The human connectome has even been suggested to be one example of a more general universality class of complex systems found in nature [125].

### 4.1. Theoretical Concepts for Graphical Models

Considering the functional organization of the brain into local interactions performing low-level information processing, called regions of interest (ROI) or modules, and long-range couplings supporting distributed information processing and providing control and high-level information fusion, brain networks form graphs intermediate between regular graphs where only nearest neighbor nodes are connected and random graphs where all nodes are connected randomly. Functional networks thus form graphs *G*(*V*, *E*), where ROI are called vertices {*V* | *v*_*n*_ : *n* = 1,…, *N*}, and long-range couplings correspond to edges {*E* | *e*_*nm*_ : *n*, *m* ∈ {1, *N*}} indicating key pathways of information processing in the brain. Vertices in the former would then be represented by single neurons or neuron pools and edges would correspond to single synapses or whole fibre tracks. Building on functional networks discussed here, vertices may correspond to either ROI or single voxels and edges to functional or effective connections between vertices. Edges are usually based on functional correlations between specific regions and afford the definition of a, somewhat arbitrary, threshold correlation to postulate an edge between two adjacent vertices. Edges may further be weighted by the related correlation coefficient. A path in a graph is a sequence of vertices connected by edges, and the length of the path is given by the number of vertices traversed. The distance between any two vertices is measured by the shortest path connecting them. The neighborhood of a vertex is given by all vertices connected to it by an edge, and the degree centrality of a vertex corresponds to the number of edges connecting to it and measures the relative importance of a vertex in a graph. Both local and global measures are frequently used to characterize the structure of functional networks.

A simple global measure of a graph is its degree distribution *P*(*V*, *E* | *k*) which measures the likelihood *ℒ*(*k*) of a vertex to have degree *k*. While for a random graph the corresponding degree distribution is a Gaussian, many complex networks show non-Gaussian degree distributions. If a given vertex shows a high-degree centrality, it is called a hub [126]. Other centrality measures of a graph are betweenness, closeness, and eigenvector centrality. The latter expresses the importance of a node in a network through the eigenvector of the adjacency matrix to the largest eigenvalue. Its *v*th component then gives the score for node *v* in the network. Degree distributions provide crucial measures of the resilience of a network to lesions or developmental defects [127–129]. A local measure of the compactness of a graph is the local clustering coefficient *C*(*v*) which measures if all directly connected neighbors *w* ∈ *U*(*v*) of node *v* are also connected to each other. It is related to the presence of the triangle motif in a network and represents the local connectivity or *cliquiness* of the node. An average over all vertices of the network yields the average cluster coefficient *C*_*G*_ which provides a global measure of the network connectivity and represents the likelihood of neighboring short connections. A related measure of connectivity is the average path length *L*_*G*_ of a graph which is the mean of all distances between any two vertices in the network. It represents the likelihood of long connections in the network and reflects the degree of integration of the given graph. Note that regular networks have large *C* and large *L*, while random networks have small *C* and small *L*. An intermediate state with high *C*, that is, many short connections, and small *L*, that is, few long connections, also exists and reflects the so-called small world properties [130]. Often these measures are normalized relative to the corresponding values of a random network with an identical number *N* of vertices. A network, exhibiting small-worldness, has *c* = *C*_*G*_/*C*_rand_ > 1 and *l*_*G*_ = *L*_*G*_/*L*_rand_ ≈ 1 leading to *s*_*G*_ = *c*_*G*_/*l*_*G*_ > 1. Given the modular nature of neuronal networks, the modularity *M* of a graph describes the degree to which a given network can be broken up into clusters of highly connected nodes, also called modules or communities, with only sparse intercluster connections. There are different definitions of modularity, and the most common one is the modularity function defined by Newman [131] which expresses the ratio of the number of existing edges in a cluster relative to the number of all possible edges in the community. Inside modules, hubs are called provincial, while hubs connecting different modules are called connector hubs. They serve to measure hierarchical structures in complex networks in as much as a hierarchical network exhibits many provincial and only few connector hubs [132]. Note that clustering coefficient, motifs, hubs, and modules describe structural aspects of a network on increasingly larger scales. Networks, which are characterized by high clustering coefficients, hence show cliques, and an abundance of hubs, and heavy-tailed degree distributions, are said to have small world properties. If their degree distributions follow a power-law behavior, they are called scale-free [133]. Such scale-free networks are especially relevant for functional network development [108]. Both anatomical connections in the brain and the synchronization networks of neurons exhibit small-world properties with exponentially truncated power-law degree distributions [9]. This topology allows for a high efficiency *F*, where (*F*_global_ ≈ *L*_*G*_^−1^, *F*_local_ ≈ *C*_*G*_), on different spatial and temporal scales, results in low wiring and energy costs and provides a high level of adaptation [134]. A small world topology thus reflects the balance between local information processing and global integration of information in the human brain. Because of this, small-world networks have special relevance for disease states. Hence, graphical models are well suited to characterize the topology of functional connectivity networks in the brain [1, 135, 136]. However, studies of the small-world properties of anatomical and functional brain networks often compare networks that differ in what the nodes represent, what kind of connectivity is measured, and what spatial and temporal scales are probed. Ioannides [137] reviewed studies of large-scale connectivity of brain networks and considered results from real-time recording techniques. He claimed that an adequate description of brain organization requires a hierarchical organization rather than single networks commonly considered. Pattern analysis methods offer a proper way to construct such hierarchies. He formulates a correspondence principle which guides the interpretation across network levels and relates nodes to anatomical entities. Recently, Wig et al. [138] provided insights into the mathematical principles underlying the incorporation of graph theory into the study of resting state brain networks. The latter display typical characteristics of complex networks, that is, they show high clustering, short path lengths, skewed degree distributions, the presence of hubs, assortative mixing, and the presence of modules [139]. Also network topology has been shown to be highly inheritable allowing prediction of cognitive function from functional similarity. Computational models are just beginning to elucidate mechanisms of complex network formation during development.

### 4.2. Some Applications of Graphical Models to Brain Networks

A couple of recent reviews deal with graph-theoretical concepts applied to complex brain networks. Reijneveld et al. [8] review older literature in the field, focussing on background knowledge in network theory and emphasizing the correlation between the structural properties of the network and its dynamics. Evidence from computational studies and neuroimaging investigations indicates that functional and anatomical connectivity of the brain show many features of small-world networks but correspond to scale-free networks only to a limited extent. Most importantly, the small-world network structure represents an optimal structure concerning rapid synchronization and information transfer, minimal wiring costs, and a balance between local processing and global integration. Most importantly, with cognitive and psychiatric disorders, these features are altered in a characteristic way. Guye et al. [140] discuss methodological developments in neuroimaging and their contribution towards an understanding of the functional organization of brain networks. They also emphasize the benefits of graph theory to elucidate the complexity of such networks and provide quantitative measures for their characterization [11]. Especially the small-world topology of these networks provides a common framework to merge structural and functional imaging as well as dynamical data. Resting state fMRI studies have provided evidence that inter-regional functional connectivity in the default mode network exhibits a small-world topology, that is, highly clustered subnetworks combined with an advanced global connectivity. Studies on scale-free topologies of such networks have remained inconclusive, however. Van den Heuvel et al. [90] consider a voxel-based approach for a model-free examination of both inter- and intraregional connectivity. From resting state fMRI recordings on healthy subjects, individual connectivity graphs were formed between all cortical and subcortical voxels which showed intervoxel functional connectivity. Graph theoretical analysis of these graphs revealed clustering coefficients much higher than for equivalent random graphs and short average path lengths. Both features reflect a small-world topology of the network. In addition, the connectivity distribution of the number of inter-voxel connections showed power-law scaling with an exponent close to 2, suggesting a scale-free topology. The results reflect a highly efficient network organization of the functionally connected brain. Voxels are mostly connected to their nearest neighbors forming clustered subnetworks. The latter are tied together by few highly connected hubs assuring a high degree of global connectivity. Partial coherence analysis has been used in [141] to also determine graphical models for functional brain connectivity. However, the outcome of such an analysis strongly depends on several factors like the degree of spectral smoothing, line and interference removal, matrix inversion stabilization, and the suppression of effects caused by side-lobe leakage. Also the combination of results from different epochs and people as well as multiple hypothesis testing may influence the results. It is shown that a diagonal upweighting of the spectral matrix can simultaneously stabilize spectral matrix inversion and suppress effects caused by side-lobe leakage. Also step-down multiple hypothesis testing helps to formulate an interaction strength. The authors claim that in this way clean connectivity plots result. Bullmore and Sporns [9] recently provided another review of graph theoretical studies of complex brain networks elucidated by diverse imaging modalities like EEG, MEG, MRI, fMRI, and DTI. Graph theoretical approaches suggest clues to the organizational principles of brain networks. The authors provide basic principles of graph theory and highlight some of the key questions to be dealt with by future developments. He and Evans [142] similarly review graph theoretical analysis of human brain networks. They reveal characteristic features of such complex networks like modularity, small world structures, scale-free structures and highly connected network hubs. These quantitative features change during development, aging, and various neurological and neuropsychiatric disorders. Furthermore, they seem to correlate with behavioral and genetic factors. Investigations with normal subjects indicate that PCC, MPFC, and IPC form the hubs of the default mode network DMN. In AD, for example, these hubs are altered in their connectivity. Miao et al. [143] studied such alterations employing Granger causality modeling and graph theoretic methods. The volunteers consisted of young adults, elder normal controls, and AD patients. Results indicated a dominant role of the PCC which showed especially wide and distinctive effects on the DMN dynamics of young adults. It was also the only hub which preserved significant causal relations to all other nodes of the DMN. MPFC and IPC exhibited disrupted causal interactions with other nodes in AD patients.

In summary, the small worldness and modularity of the structural connectivity of brain networks have been elucidated through diffusion tensor imaging (DTI) [144], diffusion spectrum imaging (DSI) [145], and cross-correlation of cortical thickness [146] and characterized by graphical models. Most studies suggested prefrontal, parietal, and temporal regions as important hubs. These regions partly overlap with the DMN and attentional networks [140]. The posterior regions, and most notably the precuneus and posterior cingulate regions, have been considered the structural core of the cerebral cortex [145]. These regions are characterized by high levels of metabolism and are the first to be involved in degenerative processes [147]. Small-worldness and modularity have also been shown from graph-theoretical characterizations of functional and effective connectivity networks elucidated mainly by fcMRI [13, 88, 148] but also with EEG and MEG techniques [135, 149]. Diverging conclusions concerning the underlying degree distributions, whether power law rather than exponentially truncated power law, of functional connectivity graphical models have been reported from such studies. These discrepancies mainly result from different methods to select proper vertices for the graphical models from the functional imaging data [90, 150, 151]. Nevertheless, potential hubs have been identified in these studies which are largely in concordance with the respective structural connectivity investigations [152] concerning the overlap with the DMN and attentional networks. Furthermore, age-related alterations in the topology of functional connectivity networks have been reported as well [153]. Such alterations are also especially critical in studies of brain pathologies. Almost all neurological and psychiatric disorders (epilepsy, schizophrenia, alzheimer's disease, dementias, autism spectrum disorders, multiple sclerosis, etc.) are characterized by network dysconnections and de-regulations [9, 12, 146].

Sofar single-graph theoretical descriptions of complex brain networks were confined to single-subject studies. Group-based brain connectivity networks have great appeal for researchers interested in gaining further insight into complex brain function and how it changes across different mental states and disease conditions. Accurately constructing these networks presents a daunting challenge given the difficulties associated with accounting for intersubject topological variability. The conventional approach has been to use a mean or median correlation network [51, 150] to embody a group of networks. Simpsona et al. [154] investigated the performance of these mean and median correlation networks. They proposed an alternative approach based on an exponential random graph modeling (ERGM) framework and compare its performance to that of the aforementioned conventional approach. They showed that the proposed ERGM approach outperforms the conventional mean and median correlation-based approaches and provides an accurate and flexible method for constructing group-based representative brain networks.

Although a number of graph theoretical characterizations of functional connectivity networks of the brain have been reported since, test-retest (TRT) reliability of topological metrics of functional brain networks has hardly been studied. Recently, Deuker et al. [155] investigated TRT reliability of graph theoretical metrics on two MEG data sets sampled from 16 volunteers at rest and during the n-back working memory task. Samples from each volunteer and each session were wavelet filtered, and mutual information (MI) between pairs of sensors was estimated in all frequency bands from *θ*-band to the *γ*-band. Undirected binary graphs were generated by thresholding the MI values, and eight global network metrics, namely, the clustering coefficient, path length, small-world property, efficiency, cost-efficiency, assortativity, hierarchy, and synchronizability, were evaluated. Reliability was assessed via intraclass correlations. Good reliability was found for most metrics during task performance and showed a positive correlation with frequency. Reliability in high-frequency bands like *β*- and *γ*-band was higher at local nodal levels than on a global level, especially in frontal and parietal regions. Metrics estimated from resting state data, thus characterizing default mode network properties, were generally less reliable. In a similar study, reproducibility of graph metrics was reported by [156]. Graph metrics were estimated for two fMRI data sets collected from 45 healthy elder volunteers. Graph metrics were compared between the two runs applying intra-class correlation coefficient (ICC) statistics and Bland-Altman (BA) plots. ICC scores were found to be high (ICC > 0.75) except for nodal degree where it was low (ICC = 0.29). Reproducibility maps, generated from these scores, showed consistently high reproducibility for global efficiency and path length across the large-scale network, while other metrics, like clustering coefficient, local efficiency, and nodal degree, achieved high reproducibility only locally in network hubs. BA plots were used in addition to test measurement reproducibility of all graph metrics. Yet another study has been performed by Wang et al. [157] considering fMRI data recorded in the resting state. Long- (>150 d) and short- (<1 h) term TRT reliability has been examined for 12 global and 6 local nodal metrics. Reliability of global metrics was generally low, threshold sensitive, and dependent on factors like scanning time interval, network membership and network type. The dependence was further modulated by the chosen node definition strategy. Reliability of local nodal metrics exhibited large variability, either with nodal degree being the most robust and reliable metric. However, nodal reliability was robust against the factors mentioned above. Additional simulations indicated that global network metrics should be very sensitive to noise, while local nodal metrics turned out to be robust against noise. The investigations shed some light onto a careful choice of analytical schemes and proper network metrics. Very recently Braun et al. [158] report another exploration of the reproducibility of graph theoretical measures of the human connectome. Measures were derived from resting state fMRI data recorded from 33 healthy volunteers. Undirected graphs were generated with the help of the anatomic-automatic labeling (AAL) atlas template. Several commonly used graph metrics, like clustering coefficient, path length, local and global efficiency, assortativity, modularity, hierarchy, and the small-worldness, were estimated and used to study the impact of confounds and strategies for confound correction. Reliability was assessed using intra-class correlation coefficients (ICC). It should be noted that data correspond to a frequency band *ν* < 0.15 Hz reflecting slow dynamics only. Overall ICCs were strongly dependent on the method employed and the metric chosen. Generally, second-order metrics, like small- worldness, hierarchy, and assortativity, tended to be more reliable than first-order metrics.

Finally, methodological issues yet to be solved have been discussed in [11]. The authors identify the following yet not completely solved problems when graphical models are to be applied to the analysis of structural, functional, or effective connectivity.

- Node selection criteria. Alternative ways of parcellation of the cortex may explain discrepancies in topological parameters extracted from graphical models [159–163]. Hence, methods of parcellation of functional imaging data of the brain need to be homogenized in order to improve consistency of parameters extracted from graphical models applied to connectivity analysis of the brain.
- Threshold selection of connection metrics. A standardization of statistical methods seems most needed for comparative studies, especially when weighted graphs are employed [164, 165].
- Relationship between anatomical structure and cognitive function. Functional connectivity between two regions of the brain does not entail a direct structural connectivity. Especially in pathological situations, more combined studies are clearly needed still [166].
